# Advancing Emergency Care With Digital Twins

**DOI:** 10.2196/71777

**Published:** 2025-04-21

**Authors:** Haoran Li, Jingya Zhang, Ning Zhang, Bin Zhu

**Affiliations:** 1 School of Public Policy and Administration Xi'an Jiaotong University Xi'an China; 2 Vanke School of Public Health Tsinghua University Beijing China; 3 School of Public Health and Emergency Management Southern University of Science and Technology Shenzhen China

**Keywords:** emergency care, digital twin, prehospital emergency care, in-hospital emergency care, recovery

## Abstract

Digital twins—dynamic and real-time simulations of systems or environments—represent a paradigm shift in emergency medicine. We explore their applications across prehospital care, in-hospital management, and recovery. By integrating real-time data, wearable technology, and predictive analytics, digital twins hold the promise of optimizing resource allocation, advancing precision medicine, and tailoring rehabilitation strategies. Moreover, we discuss the challenges associated with their implementation, including data resolution, biological heterogeneity, and ethical considerations, emphasizing the need for actionable frameworks that balance innovation with data governance and public trust.

## Introduction

**Figure 1:**
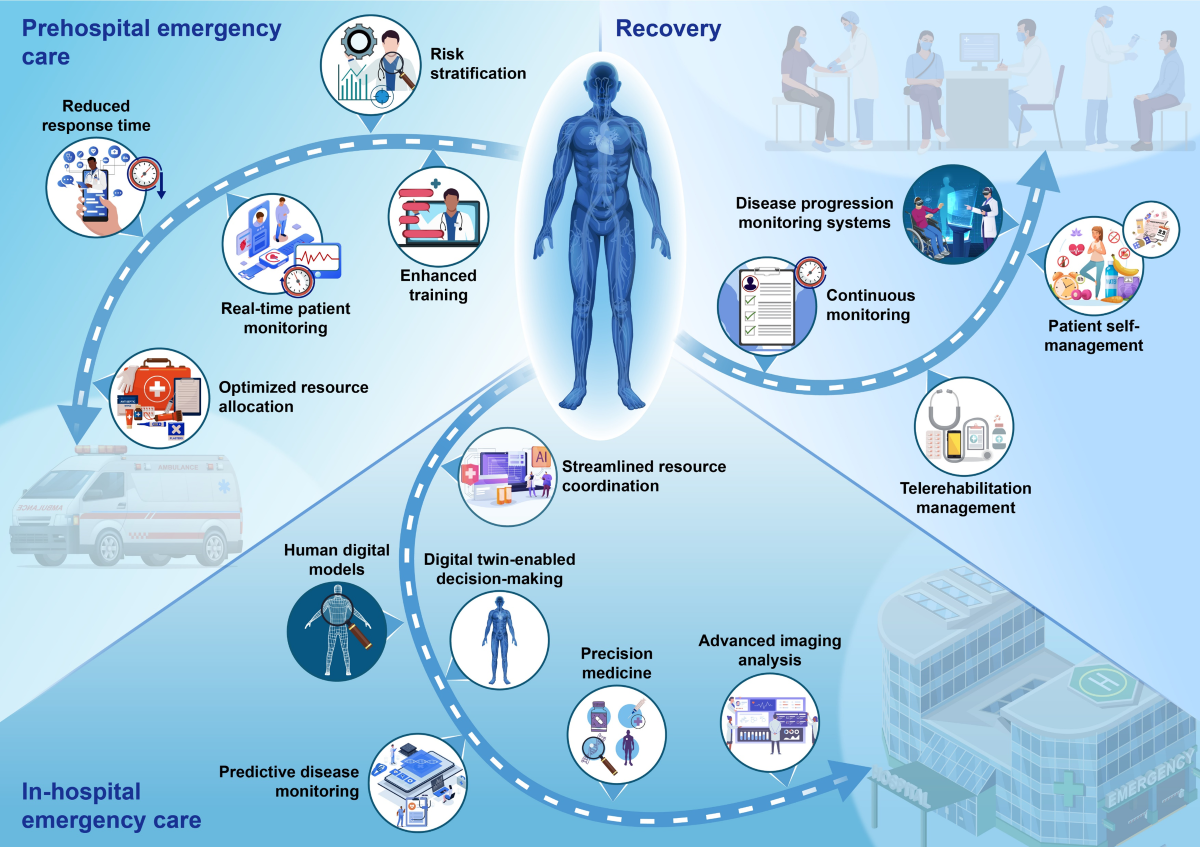
The role of digital twins in transforming emergency medicine.

The concept of digital twins—dynamic, real-time simulations of processes, systems, or environments—has garnered increasing attention across various domains, from urban planning to medical monitoring [1]. Unlike predictive analytics and artificial intelligence (AI)–driven simulations, digital twins construct dynamic mirror models of physical entities through real-time data synchronization and simulation technologies, emphasizing virtual to physical mapping and closed-loop feedback to support full lifecycle management. In contrast, predictive analytics focus on trend forecasting based on historical data, whereas AI-driven simulations concentrate on multiscenario modeling of complex systems. The advantages of digital twins lie in their real-time capabilities, dynamic optimization, and potential for cross-system collaboration, enabling a closed-loop process from monitoring to decision-making, significantly enhancing efficiency and precision [2]. Emergency care, characterized by its demand for timeliness, precision, and adaptability, provides an ideal testing ground for this transformative technology. By bridging prehospital care, in-hospital management, and recovery, digital twins promise to reshape emergency medical systems for the future (Figure 1). Textbox 1 provides an explanation of the application of digital twins in prehospital first aid, in-hospital first aid, and rehabilitation.

**The application of digital twins in prehospital first aid, in-hospital first aid, and rehabilitation.**

**Prehospital emergency care**

- Optimized resource allocation: simulation of real-time first-aid scenarios to assess urban emergency needs and spatial distribution of resources, allowing for evidence-based planning and improved efficiency in resource use
- Reduced response time: minimization of procedural delays through real-time data sharing, enabling seamless communication and critical data updates across emergency personnel and systems
- Real-time patient monitoring: leveraging of wearable devices and sensors to create dynamic health profiles, facilitating immediate assessments and predictions of patient conditions (eg, vital signs such as heart rate and blood pressure)
- Risk stratification: aggregation and analysis of patient data to provide superior accuracy in predicting acute events (eg, cardiac arrests), outperforming traditional clinical risk assessments
- Enhanced training: use of simulation-based training environments to strengthen emergency response protocols, improve decision-making, and foster team coordination under high-pressure conditions

**In-hospital emergency care**

- Advanced imaging analysis: integration of multidimensional data to overcome sample limitations in medical imaging, enabling the development of robust diagnostic models
- Precision medicine: combination of genomic, environmental, and lifestyle data for tailored risk assessments and personalized treatment plans, empowering informed clinical decision-making
- Human digital models: use of digital replicas of anatomical structures to plan surgeries and minimize risks during procedures by simulating operations beforehand
- Digital twin–enabled decision-making: facilitation of real-time analysis and expert-guided interventions (eg, cardiopulmonary resuscitation and drug administration) via remote systems, reducing disparities in emergency care accessibility
- Predictive disease monitoring: use of Internet of Things devices and wearables to continuously monitor patient status, identifying early signs of complications and supporting timely interventions
- Streamlined resource coordination: ensuring efficient resource distribution and effective communication among health care teams through unified, up-to-date patient data sharing

**Recovery**

- Continuous monitoring: use of sensors and wearable devices to track recovery progress and dynamically adjust treatment plans to optimize outcomes
- Telerehabilitation management: enabling remote monitoring of patients through digital twins, providing clinicians with tools to assess and adjust rehabilitation remotely, particularly for mobility-impaired individuals
- Patient self-management: sharing digital twin insights with patients to encourage active participation in health management through lifestyle modifications and adherence to care plans
- Disease progression monitoring systems: integration of real-time monitoring data to predict the risk of disease exacerbations or complications, thereby intervening in time and triggering early warnings, allowing physicians to adjust treatment during critical windows and improve outcomes.

## Prehospital Emergency Care

Current prehospital emergency care systems often struggle with suboptimal resource allocation due to their reliance on simplified decision-making models that fail to account for the complex, dynamic nature of urban environments [3]. Digital twins address these limitations by unifying diverse data streams into a single, adaptable model that enables real-time optimization of emergency resource allocation. These models incorporate multiple layers of data, from historical emergency call patterns to real-time traffic conditions, enabling more nuanced and effective resource deployment decisions.

Digital twins prove particularly valuable in prehospital emergency care, where time-sensitive decisions directly impact patient outcomes. By incorporating high-resolution information about building layouts, land use, population density, and traffic patterns, digital twins create actionable urban simulations that allow emergency response teams to plan and execute their operations with unprecedented efficiency. The integration of these various data sources enables more sophisticated approaches to emergency resource management than previously possible with traditional systems.

This transformation in emergency care delivery demonstrates the broader potential of digital twin technology to revolutionize urban health care systems through data-driven, predictive approaches to resource management. Moreover, wearables in ambulances provide dynamic health profiles, supporting real-time monitoring and accurate risk stratification for acute events. Simulation-based training environments can help improve emergency team readiness and coordination under high-pressure conditions.

## In-Hospital Emergency Care

In hospitals, digital twins are redefining precision medicine by integrating diverse data sources to enable robust diagnostics, personalized treatments, and streamlined workflows [4]. By synthesizing real-time patient data with historical health records and predictive algorithms, digital twins facilitate more accurate disease modeling, enhancing clinical decision-making and tailoring interventions to individual patient profiles. This data-driven approach supports the early detection of complications, optimization of treatment pathways, and improvement of patient outcomes.

Preoperative simulations represent a critical application of digital twins in surgical care. By creating highly accurate digital replicas of patient anatomy, clinicians can simulate surgical procedures, anticipate potential challenges, and refine techniques before making the first incision. These virtual rehearsals significantly reduce surgical risks, improve procedural efficiency, and enhance patient safety. Complementing this, predictive monitoring through Internet of Things (IoT) devices enables the continuous assessment of patient vital signs, offering early warnings of complications such as sepsis or cardiac arrest. These alerts allow medical teams to respond swiftly, averting adverse outcomes and saving lives.

An emerging and transformative application is the development of medical device twins (MDTs), which provide virtual counterparts for critical hospital equipment, including ventilators, defibrillators, infusion pumps, and imaging devices. MDTs are created through digital twin technology and are consistent with the actual medical device in terms of function, performance, and status. They can be updated in real time to reflect the actual operation of the medical device. These digital twins continuously monitor device performance, tracking metrics such as wear and tear, calibration accuracy, and operational efficiency. By identifying potential failures before they occur, MDTs facilitate proactive maintenance, reducing downtime and ensuring the reliability of life-saving equipment during critical emergencies.

Beyond maintenance, MDTs contribute to resource optimization. By analyzing use patterns and forecasting demand surges, these systems can dynamically allocate equipment to areas of highest need, such as during mass casualty incidents or seasonal patient influxes. This predictive capability not only prevents bottlenecks in care delivery but also enhances hospital operational efficiency, ensuring that critical resources are always available when and where they are needed.

## Recovery

In recovery, digital twins play a transformative role by enabling personalized and adaptive rehabilitation processes. These advanced systems integrate comprehensive patient data, including clinical records, biometric parameters, and lifestyle information, to develop dynamic treatment plans tailored to individual needs. Wearable devices continuously monitor patient progress in real time, providing crucial data to adjust therapies dynamically, ensuring that interventions remain effective and responsive to changes in a patient’s condition [5,6]. This real-time feedback loop facilitates precision rehabilitation, minimizing setbacks and promoting steady recovery trajectories.

Telerehabilitation further amplifies these benefits by offering remote monitoring and therapy-adjustment capabilities. Patients can receive professional guidance and care modification without frequent in-person visits, reducing logistical burdens and enhancing access to rehabilitation services. Moreover, digital twins empower patients by offering insights into their recovery progress, encouraging active engagement in health management and fostering adherence to prescribed care plans.

A significant breakthrough in the recovery phase is disease relapse prediction and early warning, a feature that underscores the predictive power of digital twins. By leveraging longitudinal patient data and advanced predictive analytics, digital twin systems can detect subtle patterns indicative of potential disease recurrence. These insights enable the generation of real-time alerts for health care providers, prompting timely interventions that can mitigate complications and prevent hospital readmissions. For instance, changes in respiratory patterns or biomarkers could signal the early stages of a relapse, allowing for preemptive adjustments in treatment strategies.

## Digital Twins in Real-World Applications

Currently, the application of digital twins in emergency care remains in its early developmental stages, with significant room for growth, yet they have already demonstrated potential in real-world scenarios. For instance, Pfizer and IBM’s collaborative Project BlueSky focuses on developing remote patient monitoring systems that use wearable devices and environmental sensors to track disease symptoms in real time, capture dynamic data, and automatically identify abnormalities for timely intervention [7]. Similarly, Digital Orthopaedics and Dassault Systèmes jointly created a clinical decision support system capable of generating patient-specific computational models to reduce trial-and-error risks and achieve precision treatment [7]. During public health crises like COVID-19, digital twins have proven effective in enhancing emergency response capabilities by simulating real-time data collection and vaccination processes to identify optimal resource allocation strategies and refine contingency planning. These examples underscore the transformative role of digital twin technology in advancing emergency care efficiency and resilience [8].

In the cases mentioned above, digital twins have demonstrated significant advantages over current public health systems. Digital public health enables optimized resource allocation, reduces waste, and enhances emergency response efficiency, and it allows real-time monitoring of patient anomalies to accelerate emergency interventions. Centered on data-driven and intelligent capabilities, these advancements markedly improve the efficiency of resource allocation, response speed, and decision-making precision in emergency care.

In summary, digital twins represent a reliable tool in the era of digital intelligence, becoming increasingly feasible in the current data-rich context. The emergency response system serves as an excellent testing ground for digital twin applications, offering significant potential across areas such as emergency resource planning, patient monitoring, and rehabilitation. However, the implementation of digital twins in the emergency domain is not a task to be completed overnight. It necessitates advancements in complementary fields, including urban modeling, wearable technology, and the IoT, to fully realize its potential. Despite their promise, the application of digital twins in emergency care faces substantial challenges. Currently, many medical digital twins remain confined to visualization, providing static representations rather than actionable tools for decision-making. For full realization, these systems must evolve beyond visualization to actionable frameworks. A second critical hurdle is data quality and resolution. Biological heterogeneity significantly influences disease progression and treatment outcomes, requiring vast, high-resolution datasets to build robust, accurate models. Lastly, ethical, privacy, and security concerns pose significant barriers to the adoption of digital twins in health care [9]. The integration of sensitive patient data with urban-level modeling raises important questions about data governance, requiring careful consideration to balance innovation with public trust. To address the aforementioned challenges, it is necessary to systematically overcome existing bottlenecks through technological optimization, privacy protection, ethical governance, and multiparty collaboration. On the privacy and security front, full lifecycle data protection must be strengthened by leveraging differential privacy and blockchain technology to anonymize sensitive information and enable traceable storage while implementing dynamic access controls to restrict unnecessary data access. Ethically and legally, clear boundaries and transparent guidelines for technology use must be established. Additionally, collaboration among governments, medical institutions, and technology companies is needed to develop unified technical standards and security evaluation systems alongside public education to build societal trust and ensure patient consent and data authorization. Only through parallel advancements in technology, regulatory frameworks, ethical constraints, and social collaboration can the safe implementation and sustainable development of digital twin technology in emergency scenarios be achieved.

## Abbreviations

AI: artificial intelligence
IoT: Internet of Things
MDT: medical device twin
